# Risks and Benefits of Preexposure and Postexposure Smallpox Vaccination^1^

**DOI:** 10.3201/eid0911.030369

**Published:** 2003-11

**Authors:** Martin I. Meltzer

**Affiliations:** * Centers for Disease Control and Prevention, Atlanta, Georgia, USA

## Abstract

This article presents a model and decision criteria for evaluating a person’s risk of pre- or postexposure smallpox vaccination in light of serious vaccine-related adverse events (death, postvaccine encephalitis and progressive vaccinia). Even at a 1-in-10 risk of 1,000 initial smallpox cases, a person in a population of 280 million has a greater risk for serious vaccine-related adverse events than a risk for smallpox. For a healthcare worker to accept preexposure vaccination, the risk for contact with an infectious smallpox case-patient must be >1 in 100, and the probability of 1,000 initial cases must be >1 in 1,000. A member of an investigation team would accept preexposure vaccination if his or her anticipated risk of contact is 1 in 2.5 and the risk of attack is assumed to be >1 in 16,000. The only circumstances in which postexposure vaccination would not be accepted are the following: if vaccine efficacy were <1%, the risk of transmission were <1%, and (simultaneously) the risk for serious vaccine-related adverse events were >1 in 5,000.

Smallpox has been identified as a weapon that may be used by a bioterrorist (*2*,*3*). Terrorist groups and even nations may have acquired stocks of smallpox produced in the former Soviet Union (*4*). As a response to this threat, the U.S. federal government has begun to produce and stockpile approximately 300 million doses of smallpox vaccine (*2*). Properly administered as a preexposure prophylactic, the vaccine is approximately 95%-98% effective. However, smallpox vaccine contains a live virus (vaccinia), and a risk for serious, vaccine-related adverse events exists (*5*,*6*). How the stockpile of smallpox vaccine should be used is much debated. Some mathematical models have suggested that, in balancing the risks of a smallpox attack against the risk for vaccine-related adverse events, only healthcare workers need be vaccinated in a preattack situation (*7*). This phase is essentially the first in the current U.S. federal government’s smallpox response plan (*8*,*9*).

Others have called for a large-scale, voluntary preexposure vaccination campaign open to the entire U.S. population (*10*,*11*). Some concur with such a position in part because they are skeptical that a postattack vaccination-based response will be adequate (*12*). A telephone survey of the U.S. population, conducted during October to December 2002, found that 61% of the respondents would accept smallpox vaccination if “. . . . offered as a precaution against terrorist attacks” (*13*). However, despite this trepidation about smallpox, the U.S. federal government‘s program to vaccinate up to 500,000 healthcare workers and first responders has found that concerns about vaccine-associated risks has caused many to question the need for preexposure vaccination (*14*–*17*). Part of this hesitancy includes questions regarding compensation for vaccine-related adverse events (*17*–*19*).

This article presents a risk-benefit model of pre- and postexposure smallpox vaccination, which will help public health officials better understand the public’s risk-benefit appraisal. Other papers have examined pre- and postsmallpox attack responses from a societal perspective (*3*,*7*). The model presented quantifies the perspective of an individual person. The model can be applied to other situations involving pre- and postexposure prophylaxis for infectious diseases (e.g., other vaccines).

## Methods

I constructed a risk-benefit model (using a standard computer-based spreadsheet; see Appendix, which balances the risks for smallpox disease against vaccine-related adverse events (vaccine-related “disease”). The general model is formulated as follows:

Net risk of disease = (risk from smallpox without preexposure vaccination) – (risk of smallpox due to vaccine failure + risk for vaccine-related adverse events from preexposure vaccination) and the precise formula is the following:  Net risk for disease = (P_R_∙P_E_∙P_T_) – [(P_R_∙P_E_ P_T_)(1–P_VEpre_) + P_SideEffect_∙P_Valuation_]  The symbols and the value for each variable are defined in the Table.

### Definitions

The term “disease” refers to case-patients with clinical symptoms caused by either smallpox or serious vaccine-related adverse events. The phrase “serious vaccine-related adverse events” includes death, postvaccinial encephalitis, and progressive vaccinia. Each serious side-effect requires medical care, such as vaccinia immunoglobulin, hospitalization, or a number of visits to a physician’s office. In 1968, the rate of postvaccinial encephalitis and progressive vaccinia among first time vaccinees ranged from approximately 0.3 to 1.2 in 100,000 for those aged 1–19 years, 0.7 to 4 in 100,000 for those <1 year of age, and 0 to 1.4 in 100,000 for those >20 years of age (20,21). As most preevent vaccinees are likely to adults, I used a rate of 1/100,000 vaccinees (P _SideEffect_, Table). Vaccine-related adverse events such as eczema vaccinatum, soreness or redness at site of vaccination, headache, and mild and temporary nausea are not considered to be serious vaccine-related adverse events in the model. A risk for eczema vaccinatum occurs in about 1 in 100,000 primary vaccinations (*20*), which can result in serious consequences requiring intensive medical care, and even (rarely) death (*6*). I thus underestimate the risk for vaccine-related adverse events, biasing the model toward acceptance of vaccination.

### Decision Criteria

If net risk for disease is >0, then the risk for disease from smallpox is greater than the risk for serious vaccine-related adverse events, and a person would chose preexposure vaccination. If the net risk for disease is <0, then the risk for serious vaccine-related adverse events is greater than the risk for smallpox, and an individual person would chose no preexposure vaccination.

### Scenarios

I use the model to evaluate the net risk for disease faced by a person who is a member of one of the following three groups: 1) The general population. The model compares the risk of being a smallpox patient before an attack is detected to the risk for serious vaccine-related adverse events from preexposure vaccination. The risk of being an actual smallpox patient is modeled by setting the risk for transmission at 1 (Table). Two populations “at risk” are modeled: a population of 9 million, representing a metropolitan area assumed to be the sole target, and the entire U.S. population of approximately 280 million. 2) The healthcare community. For a healthcare worker (HCW) who faces potential exposure to smallpox as a result of caring for a person with smallpox, the risk of contracting smallpox from the patient is compared with the risk for serious vaccine-related adverse events attributable to preexposure vaccination. 3) A smallpox investigation team. For a person who is trained to be deployed to investigate potential patients or attacks (i.e., deliberately seek out potential smallpox patients and material that may be contaminated by smallpox), the risk for contracting smallpox from the patient or other source of smallpox (e.g., aerosol, container) is compared with the risk for serious vaccine-related adverse events from preexposure vaccination. Investigation team members will take precautions to reduce risk for transmission (e.g., wear gloves, face masks, and gowns), reducing risk for transmission to an assumed 0.4 (no data exist regarding the actual reduction in risk attributable to using such barrier precautions).

**Table:** Model input variables and values used

Variable	Symbol	Values		
		Base cases	Sensitivity analyses	
Probability of attack	P_R_	1:10 –1:100,000		
No. of cases before detection of attack	X_CASE_	1,000	100,000	
General population “at risk”^a^	X_POP_	9 million or 280 million		
No. of susceptible HCW^b^	X_HCW_	100,000 or 1,000,000		
			
Probability of exposure to smallpox, for an:	P_E_			
Individual member of general populace^c^		1:9,000 or 1:280,000	1:1^j^	
Individual HCW^b^ contacting infectious person^d^		1:100 or 1;100,000	1:1^j^	
Individual member of investigation team^e^		1:2.5 or 1:5	1:1^j^	
			
Probability of transmission of smallpox, for an:	P_T_			
Individual member of general populace^f^		1.0	0.01 –0.70^j^	
Individual HCW^b^ contacting infectious person^g^		0.70	0.01 –0.70^j^	
Individual member of investigation team^h^		0.40	0.01 –0.70^j^	
			
Probability of vaccine effectiveness, preexposure	P_VEpre_	0.98^l^		
Probability of serious vaccine-related adverse events^i^	P_SideEffect_	1:100,000	1:500–1:1,000,000^j^	
Probability of vaccine effectiveness, postexposure	P_VEpost_		0.01 - 0.60^j^	
Relative individual valuation; case of smallpox : Case(s) of serious vaccine related adverse events^k^	P_Valuation_	1:1	1:35	

^a^Two populations “at risk” are modeled: a population of 9 million, representing a metropolitan area assumed to be the sole target of a smallpox attack and the entire U.S. population of approximately 280 million. Exactly how a given metropolitan area would be defined as the single target at risk is a matter of speculation. ^b^HCW, healthcare worker. ^c^Risk for exposure for member of the general populace is defined as the risk of contracting, and subsequently developing, a clinical case of smallpox before detection of the event (for individual person in general populace, P_E_ = X_CASE_/X_POP_). See text for further details. ^d^Risk of a HCW’s becoming exposed is a function of the number of cases divided by number of susceptible HCWs (for HCW, P_E_ = X_CASE_/X_HCW_). ^e^Probability of a member of an investigation team being exposed to smallpox includes the probability of being sent to a site where smallpox may be present, such as in a container. There are no data that can be used to accurately define such a risk, and the data used here are assumed. ^f^Probability of transmission of smallpox = 1 indicates that the model only considers those members from the general populace in whom a clinical case of smallpox develops. See text for further details. ^g^Probability of transmission represents when HCWs are not using any effective barrier-type protection (e.g., gloves, gowns, masks). The rate of transmission used, 0.70, is equivalent to the upper estimates of the rates of transmission to unvaccinated household members living with a smallpox patient (Appendix 1 in ref. *2*). ^h^Probability of transmission for investigation teams represents a risk after barrier-type protection is used. There are no data representing the actual reduction in risk, and the value of 0.40 is assumed. ^i^Serious vaccine-related adverse events are defined as those adverse events which require “notable” amounts of medical care, such as vaccinia immunoglobulin, hospitalization, or a number of visits to a physician’s office. The rate of 1:100,000 is derived from the number of “serious” adverse events (e.g., death, postvaccine encephalitis, progressive vaccinia) measured in 1968 among first-time adult smallpox vaccinees (*19**,**20*) ^j^These values are used to examine the risk-benefit of an individual person’s accepting smallpox vaccination, including those being revaccinated, for preexposure and postexposure scenarios. See text for further details. ^k^In the base case, it was assumed that a person would value 1 case of smallpox equal to 1 case of serious vaccine-related adverse events. However, a person may be more worried about contracting a clinical case of smallpox than experiencing vaccine-related adverse events. Thus, in the sensitivity analyses, the valuation was altered to reflect a higher valuation of a case of smallpox relative to a case of serious vaccine-related adverse events (see text for further details). ^l^ Fenner et al. (*22*) reviewed five separate studies and reported vaccine efficacy to range from approximately 91% to 97%.

For all scenarios, after an attack is detected, I assume that appropriate responses will be taken, including effective isolation of patients (*2*) and vaccination of susceptible contacts. Thus, the results only apply up to the point of discovery of the bioterrorist event.

### Sensitivity Analyses

In the model, I assume that persons considering preexposure vaccination value equally the risk for disease from either smallpox or from serious vaccine-related adverse events. In reality, a person may be more worried about contacting a clinical case of smallpox than of experiencing serious vaccine-related adverse events. The risk of dying from smallpox vaccine is approximately 1:1,000,000 vaccinees (*20*,*21*), while the death rate due to smallpox may be as high as 30% of all unvaccinated clinical cases (*23**,**24*). Using the relative risk of death, I set a comparative value of 1 case of smallpox = 35 cases of serious vaccine-related adverse events (P_Valuation_ = 1/35 = 0.02857).^2^ Other sensitivity analyses include increasing the number of cases of smallpox before detection of the attack from 1,000 to 100,000 (Table), and setting the risk for serious vaccine-related adverse events to either 1 in 10,000 or 1 in 1,000,000. The former represents the risk of experiencing probable vaccine-related myo/pericarditis, as measured during the current smallpox vaccination program among civilians (*25*). The latter is the risk, measured in the 1960s, of serious vaccine-related adverse events (e.g., postvaccinial encephalitis and progressive vaccinia) among revaccinees (*20*,*21*).

### Risk-Benefit Analysis of Postexposure Vaccination

The model can used to evaluate a person’s perspective of the risks and benefits of receiving a smallpox postexposure vaccination. I considered a person who has been exposed to somebody who may or may not have smallpox. To model such uncertainty, I set P_R_ = 1, and let P_E_ range from 1 in 10 to 1 in 100,000. I then assumed either a postexposure vaccine efficacy of 10% (P_VEpost_, Sensitivity analyses, Table) and a risk for transmission of 70% (P_T_, Sensitivity analyses, Table), or a postexposure vaccine efficacy of 60% and a risk for transmission of 35%. Additional sensitivity analyses can further vary the values for transmission and efficacy of postexposure vaccination.

I also considered the case in which a person has been exposed to a definite smallpox case (i.e., P_R_ = 1, P_E_ = 1). I examined the risk-benefit of postexposure vaccination using a range of risks for vaccine-related adverse events, from 1 in 500 vaccinees to 1 in 100,000 vaccinees. This range encompasses the risks for serious vaccine-related adverse events faced by those without any contraindications for receiving the vaccine, as well as those who would have contraindications for receiving preexposure vaccination (e.g., pregnant women, those with auto-immune diseases, HIV-positive persons). I modeled a “worst case” approach and assumed that postexposure vaccine efficacy would only be 1% (representing, for example, a delay of several days between exposure and being offered the vaccine). The risk for transmission was set at either 1% or 30%, representing possible scenarios, for example, the person considering postexposure vaccination was appropriately wearing gloves, gown, and mask in the presence of the infected person or only had a very brief contact.

### Time and the Need for Revaccinations

No data exist that record the percentage of persons in a cohort who remain free from smallpox over time (in years) after immunization against smallpox.^3^ Data from relatively small studies describe levels (titers) of vaccine-induced neutralizing antibodies for up to 50 years after vaccination or revaccination (*28*,*29*). No data correlate antibody titers to immunity from disease. Without data describing the duration of protection afforded by a single vaccination, the current model does not consider the need for additional revaccinations over time. Thus, the results for this model only apply to the primary vaccinations. The model does not discount risk over-time, although some evidence exists that persons have a high discount rate for personal health issues (*30*).

## Results

**Figure 1:**
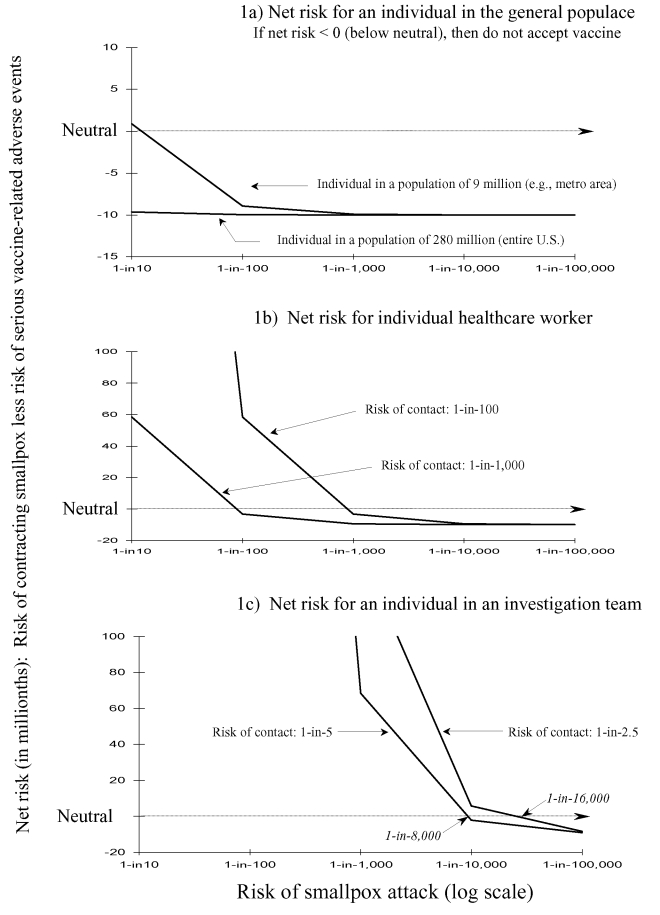
Risk-benefit analyses for individual persons evaluating the risk for smallpox versus the risk for serious smallpox vaccine-related adverse events: three scenarios. If the net risk is >0 (above neutral), then a person will accept preexposure vaccination. If the net risk is <0 (below neutral), then the person would not accept preexposure vaccination. Part a considers a person who is either a member of a population of 9 million, representing a metropolitan area assumed to be the sole target of a smallpox attack and the entire U.S. population of approximately 280 million. In part b, the risk for contact by an individual healthcare worker is a function of probability of contact x probability of transmission (P_E_ x P_T_, see Table and text for further details). In part c, investigation team members are assumed to take precautions against transmission (e.g., wear gloves, face masks, and gowns) to reduce risk to 0.4 (no data of the actual reduction in risk due to using such barrier precautions). Threshold values of risk for smallpox attack, when net risk = 0 (neutral), are rounded to the nearest 1,000. All three parts present data calculated on the basis of an attack that initially causes 1,000 cases before detection of the attack. See Table and text for other assumptions.

When these decision criteria are used for a person in a general population of 280 million, the risk for serious vaccine-related adverse events is greater than the risk for smallpox (Figure 1a). This is true even if the risk for a smallpox attack is 1 in 10. An individual person would therefore decline preexposure vaccination. Only when the population at risk is limited to 9 million and the risk for attack approaches 1 in 10, does the risk for smallpox outweigh the risk for serious vaccine-related adverse events, indicating that the person would accept vaccination (Figure 1a). For a HCW to accept preexposure vaccination, the risk for contact with an infectious case of smallpox must be >1 in 100 and the probability of an attack causing 1,000 cases must be >1 in 1,000 (Figure 1b). If the risk for contact drops to 1 in 1,000, then the risk for attack must be >1in 100 to accept vaccination (Figure 1b). For a member of an investigation team, if the anticipated risk for contact is 1 in 2.5 and the risk for attack is >1 in 16,000, then a team member would accept preexposure vaccination (Figure 1c). If the risk for contact drops to 1 in 5, then the risk for attack must increase to >1 in 8,000 for the person to accept preexposure vaccination (Figure 1c).

### Sensitivity Analyses

**Figure 2:**
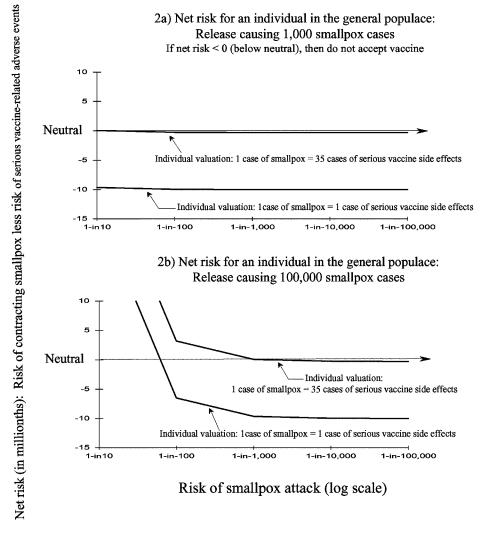
Sensitivity analyses: impact of altering a person’s value of a case of smallpox relative to a case of serious smallpox vaccine-related adverse events. If the net risk is >0 (above neutral), then a person will accept preexposure vaccination. If the net risk is <0 (below neutral), then the person would not accept preexposure vaccination. Both parts show the impact of altering a person’s valuation of a case of smallpox relative to a case of serious vaccine-related adverse events. Part a shows the net risks for an individual person’s considering preexposure smallpox vaccination with an attack causing clinical cases of smallpox to develop in 1,000 persons. Part b shows the net risks for a person when an attack causes clinical cases of smallpox to develop in 100,000 persons (see text for further details).

If a member of the general population of 280 million were to equate 1 case of smallpox to 35 cases of serious vaccine-related adverse events, they would accept preexposure vaccination only if the risk for a smallpox attack approached 1 in 10 (Figure 2a). However, if the risk for attack is assumed to be 1 in 100, then the person would have to equate 1 case of smallpox to 290 cases of serious vaccine-related adverse events to accept preexposure vaccination (data not shown). If a person assumes both that the initial attack would cause 100,000 cases before detection and that 1 case of smallpox is equivalent to 35 cases of serious vaccine related adverse events, then the risk for attack would have to be >1 in 1,000 to accept preexposure vaccination (Figure 2b).

Assuming a risk for serious vaccine-related adverse events of 1 in 10,000 (*25*) and the same values used to produce Figure 1a, a person in a population of 9 million would not accept vaccination even if the risk for attack were 1 in 2. When the same risk for adverse events is used in considering the scenarios evaluated in Figure 2b (100,000 cases before detection, valuation of 1 case smallpox = 35 cases of vaccine-related adverse events), the risk for attack would have to be >8 in 1,000 before accepting vaccination (results not shown).

### Revaccination

For a person in a population of 280 million who is considering preexposure revaccination with a risk for serious vaccine-related adverse events of 1 in 1,000,000, even at a 1 in 10 risk for smallpox attack, the net risk is <0, and the decision criteria would indicate not accepting revaccination (scenario assumed 1,000 smallpox cases before discovery of the attack, and setting P_Valuation_ = 1:1). In the same scenario, if P_Valuation_ = 1:35, then the risk for a smallpox attack would have to be >1 in 125 for a person to accept revaccination. For a HCW to accept preexpsoure vaccination, the risk for attack would have to be >1 in 700 (risk for contact = 1-in-1,000; P_Valuation_ = 1:1; revaccination P_SideEffect_ = 1-in-1,000,000). If the HCW assumed that the risk for contact increased to 1 in 100, then the risk for attack would have to be >1 in 7,000 in order to accept revaccination.

### Postexposure Vaccination

**Figure 3:**
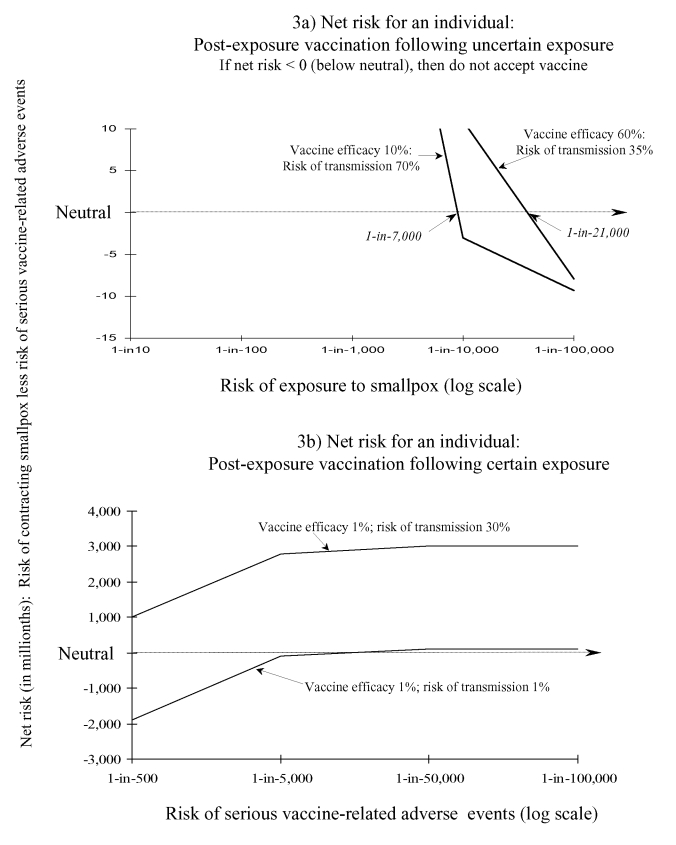
Risk-benefit analyses for persons considering postexposure smallpox vaccination: two scenarios. If the net risk is >0 (above neutral), then the person will accept postexposure vaccination. In the net risk is <0 (below neutral), then the person would not accept postexposure vaccination. Part a shows the net risk for postexposure smallpox vaccination for a person who has been exposed to somebody who may or may not have smallpox (i.e., the exposure is uncertain). Threshold values of risk for exposure to smallpox, when net risk = 0 (neutral), are rounded to the nearest 1,000. Part b shows the net risk for an individual person who has been exposed to a definite smallpox case (see text for further details).

After uncertain exposure to smallpox (e.g., contact with a person who may or may not be infectious with smallpox), the decision criteria would indicate acceptance of postexposure vaccination if the risk for exposure is thought to be >1 in 21,000; the risk for transmission is assumed to be 35%, and efficacy of postexposure vaccine is 60% (Figure 3a). If the risk for transmission is assumed to be 70%, but postexposure vaccine efficacy only 10% (e.g., postexposure vaccination offered several days after potential exposure), vaccination would be accepted only if the risk for exposure is assumed to be >1 in 8,000 (Figure 3a). If postexposure vaccine efficacy were set at 98%, and risk for transmission at 70%, then risk for actual exposure to smallpox would have to be >1 in 69,000 in order to accept postexposure (data not shown).

For persons who have had a definite exposure to smallpox, the only time that postexposure vaccination would not be accepted is if vaccine efficacy was <1%, risk of transmission was <1%, and the risk for serious vaccine-related adverse events were >1 in 5,000 (Figure 3b). In the same scenario, if the risk for transmission were 30%, postexposure vaccination would accepted even if risk for serious vaccine-related adverse events were 1 in 500 (Figure 3b).

Figures 1 and 2 show that the single most influential variable impacting the net risk for disease, and therefore the decision to accept preexposure vaccination, was the probability of attack of smallpox. For persons in the general population, the second most important variable is the valuation of one case of smallpox relative to cases of serious vaccine-related adverse events (P_Valuation_). For a HCW or a member of an investigation team, the second most important variable was the risk for contact with a smallpox patient or infectious material.

## Conclusions

The model suggests that most persons in the general population would not accept preexposure smallpox vaccination. Increasing the risk for vaccine-related adverse events (e.g., including the risk for eczema vaccinatum and vaccine-related myo/pericarditis) moves all the graph lines in Figures 1 and 2 downward. This supposition increases the likelihood of not accepting preexposure vaccination. These results and conclusions are not unique. In 1971, some argued that the risks for routine childhood smallpox vaccination in the United States outweighed the risks of contracting a case of smallpox (*4*,*31*,*32*). These arguments influenced the 1971 recommendation to stop routine childhood immunization against smallpox in the United States (*33*). The studies and arguments influencing the decision took an implicit societal perspective, while this study considers the perspective of the individual person.

For an individual healthcare worker, the decision to accept preexposure vaccination hinges almost as much on the assessment of risk for contact (before discovery of attack) as on the assessment of risk of attack. In the mid-Atlantic states of New Jersey, New York, Pennsylvania (New Jersey, New York, and Pennsylvania), approximately 440 general hospitals exist; 83% operate an emergency room (*34*). These hospitals are staffed by approximately 18,000 full-time equivalents (FTEs) physicians and dentists, 160,000 nurse FTEs (in NY 1 nurse FTE = 1.13 persons), 24,144 trainees and approximately 430,000 “other salaried” staff, for a total staff of approximately 650,000 (*34*,*35*). If one assumes that 10% work in the emergency rooms, 65,000 hospital staff in New Jersey, New York, and Pennsylvania are vulnerable to infection before a smallpox attack is detected. Further assume that an attack causes 1,000 smallpox cases confined to the New Jersey, New York, Pennsylvania area. By days 7–8 postinfection, <20% of those will proceed to the prodrome and rash stages (*1*,*2*), perhaps causing medical care to be sought. Blendon et al. (*13*) reported that 52% of survey respondents stated that they would go to their own family doctor if they thought they had smallpox, with 42% stating that they would go to a hospital emergency room. Thus, approximately 100 patients (1,000 x 20% early cases x 50% to hospital) might seek medical care at a hospital in the first 7–9 days after infection.

The healthcare workers in emergency rooms therefore face a risk for exposure to an infectious smallpox patient of change to <1 in 600 (65,000/100). If one assumes a risk for transmission of 70%, the risk of contracting smallpox is almost 1 in 1,000. The many part-time and temporary workers in a hospital further reduces this risk ratio. Even if one patient can potentially infect up to 10 healthcare workers in a hospital setting (*36*), the risk is still 1 in 65. Note that the risk for exposure is not confined to medical doctors or nurses. Many members of a hospital staff, such as those working in housekeeping and maintenance, are at risk of coming into contact with an infectious patient.

Figure 3a may suggest to some that almost any exposure to a possible case of smallpox, such as coming into contact with a person with an unexplained rash, would warrant immediate postexposure vaccination (e.g., before laboratory confirmation that patient with unknown rash actually had smallpox). However, postexposure vaccination given within 7 days after exposure reduces the risk of a clinical case of smallpox developing to approximately 2% compared with 79% among those never vaccinated (*37*). If vaccination is delayed up to 10 days postexposure, then the risk for smallpox may be reduced just 22% (from 96% among those never vaccinated to 75% among those vaccinated within 10 days postexposure) (*38*).^4^ A more compelling conclusion from Figure 3a is that if, by day 6 postexposure, the type of exposure cannot be accurately determined and a person could have been exposed to smallpox (i.e., risk for exposure is >1 in 21,000), then the person would use the decision criteria to accept postexposure vaccination.

The biggest problem in interpreting the results from the model is understanding how a person will actually value risks and events. Valuing risks depends on understanding probabilities, which are often difficult to explain (*41*). Even the type of visual aid used to explain risk can make a difference in valuation (*42*). Merely stating a number (e.g., 1 in 10,000) is often not sufficient. A person’s valuation of the risks and benefits of vaccination may include factors not explicitly defined in the model. A person may accept preexposure vaccination, for example, as an attempt to reduce potential risk for smallpox to family and friends and even out of a sense of duty to society in general. The valuation of a case of smallpox relative to a case of serious vaccine-related adverse events is a proxy for valuing a person’s contribution to family, friends, and society.

Public health planners and medical care providers should appreciate the extent that an individual acceptance or rejection of smallpox vaccination depends on valuation of risk and benefits. A person’s risk aversion is not completely explained by numerical analyses (*43*,*44*). A person’s valuation of risk depends on a variety of factors, including a sense of control, degree of trust of the source providing the data, the newness of the risk, and even the passage of time (*41*,*45*). Note that time and information may not alter the actual risk faced, but a factor such as new information (e.g., reported cases of vaccine-related adverse events) could alter the perception and valuation of risk. Accurately predicting the direction and extent of a change in valuation attributable to, for example, new information may not be possible. Public health officials, however, must always be prepared to explain how the new information alters the risks involved. Explaining a given risk, and how a new development may impact that risk, will likely require more than just a single numerical statement.

## Supplementary Material

Appendix Risks and Benefits of Pre- and Postexposure Smallpox Vaccination

## Footnotes

2 Assume that only a single metropolitan population of 9 million is at risk from an initial attack, and the initial attack results in 1,000 cases before discovery. For a person in that population, the risk for death from smallpox is approximately 33 times greater than the risk for death from the smallpox vaccine [smallpox risk for death/vaccine-related risk for death = (1,000 cases/ 9 million x 0.3)/0.000001]. For a person in a population of 280 million, the risk of dying from smallpox in the initial 1,000 cases is approximately equal to the risk for death from the vaccine.

3 In data reported by Rao from Madras, India (Figures 17/1 and 17/3 [*23*]), among the unvaccinated, approximately 80% of all cases of smallpox occurred in children <10 years of age. A distinct shift in age of the case-patients occurred among the vaccinated, with <10% of cases occurring in children <10 years of age, 19% of cases occurring in children 10–19 years, and 46% of cases occurring in persons 20–30 years of age. Rao did not report at what age most of those vaccinated received vaccine (a likely hypothesis would be before 2 years of age). Further complicating the analysis of such data is the fact that many persons in Madras received more than one smallpox vaccination (Figure 17/5, [*23*]). A similar age-shift in occurrence of cases among the vaccinated can be discerned from the data reported by Hanna (*24*) from Liverpool, England, in 1902–03. The data from both Rao (*23*) and Hanna (*26*) further indicate that even a 20-year-old vaccination may reduce the severity of disease. The risk for death is markedly reduced 20–30 years postvaccination (*23*,*26*,*27*).

4 In addition to the estimates quoted in the main text, Rao et al. (*39*) found that successful postexposure vaccination reduced, on average, the rate of smallpox among contacts by approximately 38% (from 48% among unvaccinated to 30% among postexposure vaccinees). Dixon (*40*) reported that in a group of 59 contacts under 5 years of age “. . . approximately half of those who had a successful vaccination after contact developed disease.” The wide variations in reports of the degree of protection afforded by postexposure vaccination are probably due to a number of reasons, including small sample sizes and difficulty in determining when exposure and potential transmission actually took place.
